# Association Between Serum Ferritin and Glycated Hemoglobin: Experience From the Biochemistry Laboratory of Mohammed VI University Hospital, Oujda

**DOI:** 10.7759/cureus.113752

**Published:** 2026-07-31

**Authors:** Rhoubi Asmae, Faiz Ismail, Joudar Fatima-zahra, Elhoucine Sebbar, Dounia El Moujtahide, Mohammed Choukri

**Affiliations:** 1 Medical Biology, Mohammed VI University Hospital Center of Oujda, Oujda, MAR; 2 Hematology, Mohammed VI University Hospital Center of Oujda, Oujda, MAR; 3 Laboratory Medicine, Mohammed VI University Hospital Center of Oujda, Oujda, MAR; 4 Biochemistry, Mohammed VI University Hospital Center of Oujda, Oujda, MAR

**Keywords:** diabete mellitus, ferritinemia, glycated hemoglobin (hba1c), glycemic control, insulin resistance, serum ferritin

## Abstract

Background and objectives

Serum ferritin, a commonly used indicator of body iron stores, has been associated with glycemic status in patients with diabetes mellitus. However, the relationship between serum ferritin concentrations and glycemic control remains controversial. This study aimed to evaluate the association between serum ferritin concentrations and glycated hemoglobin (HbA1c) levels in patients with diabetes mellitus and to examine this relationship according to diabetes type and glycemic control in patients with type 2 diabetes (T2D).

Methods

A retrospective cross-sectional study was conducted at the Biochemistry Laboratory of Mohammed VI University Hospital in Oujda, Morocco. A total of 1,412 patients with diabetes who underwent simultaneous serum ferritin and HbA1c testing between January and December 2023 were included. Patients were classified as having type 1 diabetes (T1D) or T2D. Patients with T2D were further categorized according to glycemic control. HbA1c values were summarized as mean ± standard deviation, whereas serum ferritin concentrations were expressed as median (interquartile range) because of their non-normal distribution. The association between serum ferritin and HbA1c was assessed using Spearman's rank correlation coefficient. Ferritin concentrations across glycemic control groups were compared using the Kruskal-Wallis test.

Results

The study included 214 patients with T1D and 1,198 with T2D. No significant association was observed between serum ferritin concentrations and HbA1c levels in patients with T1D (ρ=0.017, p=0.806). In contrast, a strong positive association was found in patients with T2D (ρ=0.871, p<0.001). Serum ferritin concentrations increased significantly across worsening categories of glycemic control in T2D (Kruskal-Wallis test, p<0.001).

Conclusion

Serum ferritin concentrations were strongly associated with HbA1c levels and worsening glycemic control in patients with T2D but not in those with T1D. Although these findings support an association between serum ferritin concentrations and glycemic control, the cross-sectional design does not allow causal inference. Prospective studies are warranted to further clarify the clinical significance of this association.

## Introduction

As one of the most prevalent chronic endocrine disorders worldwide, diabetes mellitus poses a major public health challenge owing to its increasing incidence and chronic complications [1]. Glycated hemoglobin (HbA1c) is a well-established biomarker generated by the non-enzymatic glycation of the N-terminal valine of the hemoglobin β-chain in the presence of chronic hyperglycemia [2]. By reflecting the mean glycemic level over the preceding two to three months, HbA1c provides a dependable measure of metabolic control and therapeutic effectiveness in individuals with diabetes [2]. Recent evidence has highlighted a close relationship between iron metabolism and glucose homeostasis. This association was first recognized in hereditary hemochromatosis and has subsequently been investigated in several conditions, including β-thalassemia and type 2 diabetes (T2D) [3-7]. Although the underlying mechanisms remain incompletely understood, experimental and clinical studies suggest that alterations in iron metabolism may contribute to oxidative stress, impaired insulin signaling, and disturbances in glucose homeostasis [7]. Several studies have reported an association between serum ferritin concentrations and diabetes; however, the clinical significance of elevated ferritin levels in assessing glycemic imbalance and monitoring disease progression in patients with T2D remains uncertain. Likewise, the potential role of serum ferritin as an easily accessible biomarker of metabolic dysregulation has not yet been fully established, and published findings remain inconsistent.

In this context, investigating the relationship between serum ferritin levels and HbA1c may provide additional insight into the interaction between iron metabolism and glycemic control. Therefore, the primary objective of the present study was to assess the association between serum ferritin concentrations and HbA1c levels in patients with T2D. A secondary objective was to compare serum ferritin concentrations across categories of glycemic control defined according to HbA1c values.

## Materials and methods

This retrospective cross-sectional study was conducted at the Biochemistry Laboratory of Mohammed VI University Hospital, Oujda, Morocco, using laboratory data collected between January 1 and December 31, 2023.

During the study period, approximately 5000 HbA1c test records were identified through the Laboratory Information System (LIS). After applying the eligibility criteria, 1412 adult patients (aged 18-80 years) were included in the final analysis. Eligible participants had either type 1 diabetes (T1D) or T2D and had both serum ferritin and HbA1c measured on the same day. Patients with incomplete laboratory records or missing serum ferritin or HbA1c measurements were excluded.

Data were extracted retrospectively from the Laboratory Information System (LIS) of the Biochemistry Laboratory. Demographic variables included age and sex, while laboratory variables included serum ferritin and HbA1c concentrations.

Serum ferritin was analyzed as a continuous variable. HbA1c (%) was used as an indicator of glycemic control and was analyzed both as a continuous variable and as a categorical variable. For subgroup analyses, patients with T2D were classified into three glycemic control categories according to the American Diabetes Association (ADA) recommendations: good glycemic control (HbA1c<7%), moderate glycemic control (HbA1c 7 to <9%), and poor glycemic control (HbA1c ≥9%).

Serum ferritin concentrations were measured using a chemiluminescent microparticle immunoassay (CMIA) on the ARCHITECT i8200 analyzer (Abbott Diagnostics, Abbott Park, IL, USA). HbA1c was measured by high performance liquid chromatography (HPLC) using the ADAMS HA-8180V analyzer (ARKRAY Inc., Kyoto, Japan).

Statistical analyses were performed using IBM SPSS Statistics for Windows, Version 26 (Released 2019; IBM Corp., Armonk, New York, United States). The normality of continuous variables was assessed using the Shapiro-Wilk test. Variables with a normal distribution were expressed as mean ± standard deviation (SD), whereas variables with a non-normal distribution were presented as median and interquartile range (IQR). Accordingly, HbA1c values were summarized as mean ± SD because they followed a normal distribution, while serum ferritin concentrations were reported as median (IQR) because they showed a non-normal distribution.

The association between serum ferritin concentrations and HbA1c levels was assessed separately in patients with T1D and T2D using Spearman's rank correlation coefficient. HbA1c was analyzed as a continuous variable for correlation analyses and categorized according to the ADA criteria for subgroup analyses in patients with T2D. Among patients with T2D, differences in serum ferritin concentrations across the three glycemic control categories were evaluated using the Kruskal-Wallis test. When appropriate, post hoc pairwise comparisons were performed using the Dunn Bonferroni correction. Comparisons of serum ferritin concentrations according to sex were performed using the Mann Whitney U test. All statistical tests were two sided, and a p-value<0.05 was considered statistically significant.

## Results

During the study period, approximately 5,000 HbA1c test records were identified through the LIS. After applying the eligibility criteria, 1,412 patients were included in the final analysis, comprising 214 patients with T1D and 1,198 patients with T2D.

The mean age of patients with T2D was 64.8 ± 10.9 years, compared with 29.6 ± 7.6 years in patients with T1D. Female patients predominated in both groups, with a female-to-male ratio of 0.68 in the T2D group and 0.57 in the T1D group. The mean HbA1c level was 7.5 ± 2.0% in T1D patients and 7.5 ± 1.7% in T2D patients. Median serum ferritin concentrations were 45.8 ng/mL (IQR: 15.9-114.6) in patients with T1D and 472.4 ng/mL (IQR: 100.6-1038.0) in patients with T2D.

**Figure 1:**
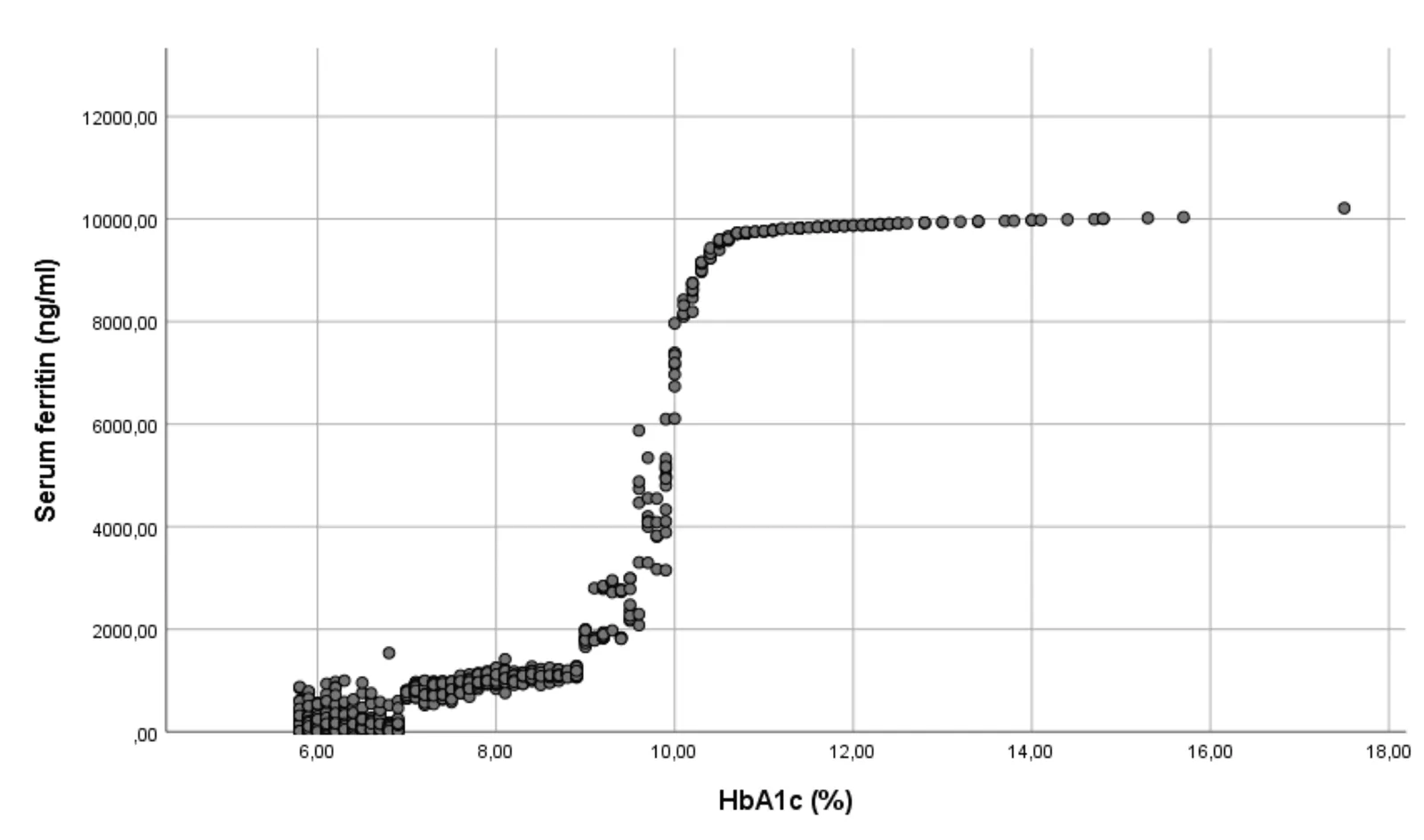
Scatter plot showing the association between serum ferritin concentrations and HbA1c levels in patients with T2D Each point represents one patient with type 2 diabetes mellitus (T2D). The x-axis represents HbA1c (%) and the y-axis represents serum ferritin concentrations (ng/mL). The figure illustrates the positive association between serum ferritin concentrations and HbA1c levels observed in this study (Spearman's ρ=0.871, p<0.001).

No statistically significant association was observed between serum ferritin and HbA1c in patients with T1D (Spearman's ρ=0.017, p=0.806). In contrast, among patients with T2D, a strong positive monotonic association was observed between serum ferritin and HbA1c (Spearman's ρ=0.871, p<0.001; Figure 1, Table 1).

**Table 1:** Association between serum ferritin concentrations and HbA1c levels according to diabetes type T1D: type 1 diabetes; T2D: type 2 diabetes; HbA1c values are expressed as mean t standard deviation (SD). Serum ferritin concentrations are presented as median (interquartile range, IQR). Associations between serum ferritin and HbA1c were assessed using Spearman's rank correlation coefficient.

Diabetes type	N	Mean HbA1c ± SD (%)	Median ferritin (IQR) (ng/mL)	Spearman's p	p-value
T1D	214	7.5 ± 2	45.8 (15.9-114.6)	0.017	0.806
T2D	1198	7.5 ± 1.7	472.4 (100.6-1038.0)	0.871	<0.001

Patients with T2D were further classified according to glycemic control into three categories: good control (HbA1c<7%), moderate control (HbA1c 7 to <9%), and poor control (HbA1c ≥9%).

Among the 1,198 patients with T2D, 586 (48.9%) had good glycemic control, 406 (33.9%) had moderate glycemic control, and 206 (17.2%) had poor glycemic control.

**Figure 2:**
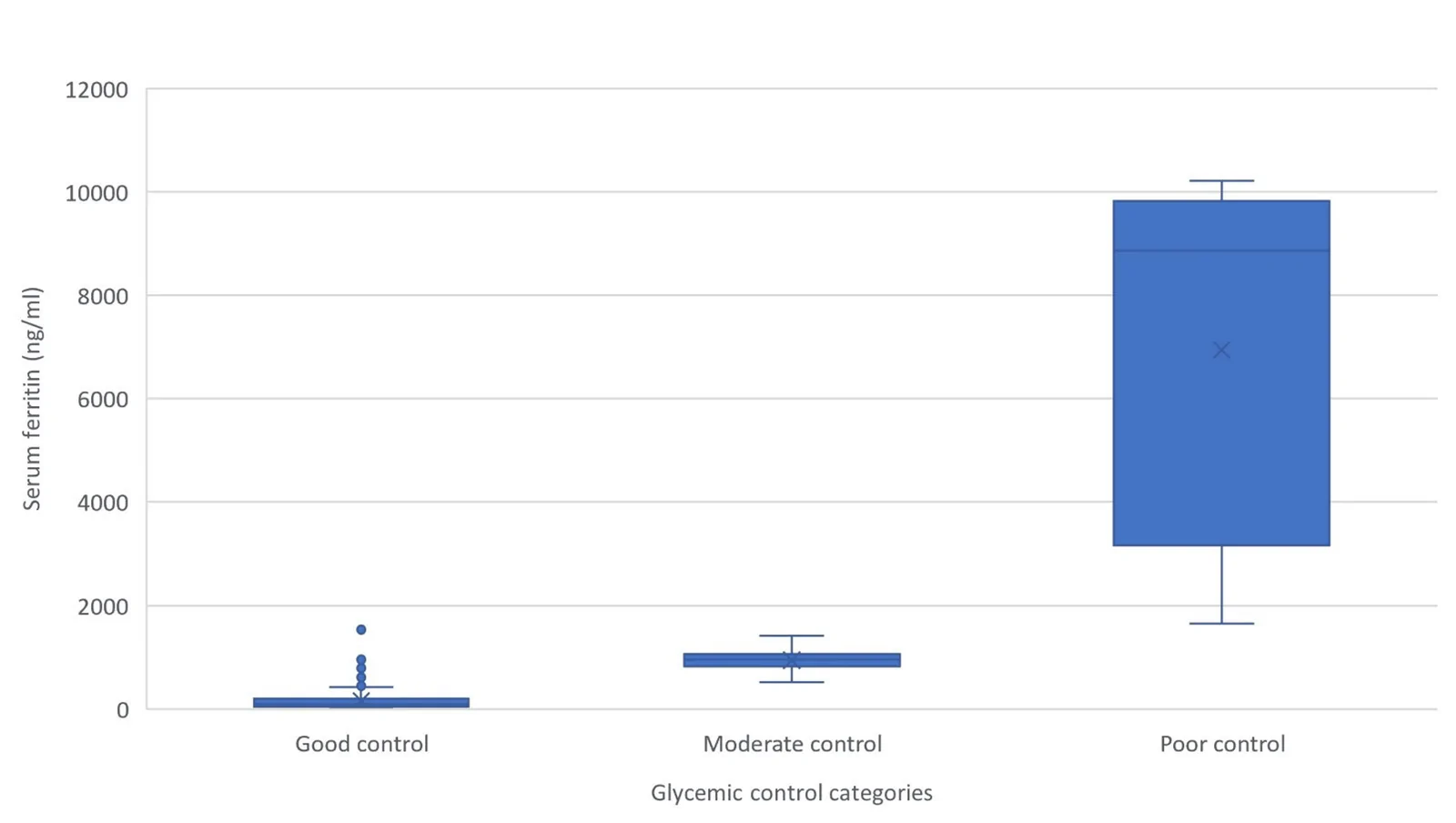
Box-and-whisker plot showing the distribution of serum ferritin concentrations according to glycemic control categories in patients with type 2 diabetes (T2D) Boxes represent the interquartile range (IQR), the horizontal line within each box represents the median, and whiskers indicate the data spread. Individual points represent outliers. Serum ferritin concentrations increased significantly across worsening glycemic control categories (Kruskal-Wallis test: H=986.574, df=2, p<0.001).

Median serum ferritin concentrations increased progressively across the three glycemic control categories, from 124.3 ng/mL (IQR: 51.6-303.0) in the good-control group to 866.4 ng/mL (IQR: 341.7-1027.2) in the moderate-control group and 3358.2 ng/mL (IQR: 2352.9-3969.1) in the poor-control group (Figure 2).

Comparison of serum ferritin concentrations among the three glycemic control groups using the Kruskal-Wallis test demonstrated a statistically significant difference (H=986.574, df=2, p<0.001; Table 2).

**Table 2:** Comparison of serum ferritin concentrations according to glycemic control categories in patients with type 2 diabetes (T2D) Serum ferritin concentrations differed significantly among the three glycemic control categories (Kruskal-Wallis test: H=986.574, df=2, p<0.001). HbA1c values are expressed as mean ± standard deviation (SD), and serum ferritin concentrations as median (interquartile range, IQR).

Glycemic control	N	Mean HbA1c ± SD (%)	Median ferritin (IQR) (ng/mL)
Good control (HbA1c<7%)	586	6.2 ± 0.33	124.3 (51.6-303)
Moderate control (HbA1c 7 to <9%)	406	7.79 ± 0.57	866.4 (341.7-1027.2)
Poor control (HbA1c ≥9%)	206	10.66 ± 1.48	3358.2 (2352.9-3969.1)

No statistically significant difference in serum ferritin concentrations was observed between male and female patients with T2D (Mann-Whitney U=161613.5, p=0.055).

## Discussion

Our study, which included 1,412 patients, demonstrated a strong positive association between serum ferritin concentrations and HbA1c levels in patients with T2D, whereas no significant association was observed in patients with T1D. Furthermore, serum ferritin concentrations increased significantly across categories of worsening glycemic control in T2D. These findings are consistent with previous studies reporting an association between elevated serum ferritin concentrations and poorer glycemic control in patients with T2D [3-7].

Several studies have reported significantly higher serum ferritin concentrations in patients with T2D than in healthy individuals and have suggested that ferritin may reflect alterations in iron metabolism associated with metabolic dysfunction [3-7]. Experimental and clinical studies have proposed several mechanisms that could explain this association. Disturbances in iron metabolism may contribute to oxidative stress, impaired insulin signaling, and increased hepatic glucose production, thereby promoting insulin resistance and impaired glucose homeostasis [8,9]. However, these mechanisms remain incompletely understood, and the cross-sectional design of the present study does not allow causal inferences.

Our findings are in agreement with those reported by Khalil et al. [3], Elimam et al. [4], Gandhi et al. [5], Ford et al. [6], and Raj and Rajan [7], all of whom described positive associations between serum ferritin concentrations and glycemic control indices in patients with T2D. In contrast, Kaye [10] reported a negative correlation between ferritin and glycemic parameters, whereas Sharifi and Sazandeh [11] found no significant association. These discrepancies may reflect differences in study populations, ethnicity, inflammatory status, laboratory methods, and study design.

Previous epidemiological studies have suggested that elevated serum ferritin concentrations may be associated with an increased risk of T2D and insulin resistance [12]. Likewise, studies conducted in African and Asian populations have reported higher serum ferritin concentrations in patients with diabetes than in healthy controls [13-16]. Nevertheless, serum ferritin should be interpreted cautiously because it is also an acute-phase reactant and may increase in inflammatory, infectious, hepatic, or other clinical conditions independently of body iron stores.

In the present study, serum ferritin concentrations increased progressively across the three categories of glycemic control in patients with T2D. Patients with poor glycemic control exhibited the highest ferritin concentrations, and the difference among the three groups was statistically significant according to the Kruskal-Wallis test. These findings further support the existence of an association between serum ferritin concentrations and worsening glycemic control, although they do not establish a causal relationship.

Contrary to some previous reports, no statistically significant difference in serum ferritin concentrations was observed between male and female patients in our study (Mann-Whitney U test, p=0.055). This discrepancy may be explained by differences in study populations, sample characteristics, or residual confounding factors.

Limitations

Several limitations should be acknowledged. First, the retrospective cross-sectional design precludes establishing a causal relationship between serum ferritin concentrations and glycemic control. Second, this was a single-center study, which may limit the generalizability of the findings. Third, serum ferritin is an acute-phase reactant, and complementary inflammatory markers such as C-reactive protein (CRP), interleukin-6 (IL-6), and tumor necrosis factor-α (TNF-α) were not available. Consequently, residual confounding due to unrecognized inflammatory conditions cannot be excluded. Finally, information on potential confounding factors such as obesity, medications, liver disease, or other metabolic comorbidities was not available because the analysis was based exclusively on laboratory records.

## Conclusions

In conclusion, serum ferritin concentrations were strongly associated with HbA1c levels in patients with T2D but not in patients with T1D. Serum ferritin concentrations also increased significantly across categories of worsening glycemic control in T2D. Although these findings support an association between iron metabolism and glycemic control, they do not establish causality. Prospective multicenter studies incorporating inflammatory and metabolic biomarkers are warranted to clarify the clinical significance and underlying mechanisms of this association.
